# A systematic review of the experience of treatment burden of digital health for military personnel in primary healthcare.

**DOI:** 10.12688/healthopenres.13599.1

**Published:** 2024-03-08

**Authors:** Paul Erhahiemen, Catherine A. O'Donnell, Katie Gallacher, Barbara I. Nicholl

**Affiliations:** 1 General Practice & Primary Care, School of Health and Wellbeing, University of Glasgow, Glasgow, Scotland, G12 8TB, UK

**Keywords:** Digital health, artificial intelligence, digitalisation, primary care, military, service personnel, treatment burden, patient experience

## Abstract

**Background:**

Digital Health (DH) integrates digital technologies into healthcare to increase efficiency and improve patient experiences, benefiting both primary care and military healthcare systems. However, it raises concerns about the potential shift of healthcare responsibilities onto patients, creating workloads or treatment burdens that affect care, adherence, equity, and resource allocation. It is critical to assess this in the military context to enhance patient-centred care and outcomes.

**Objective:**

To understand military personnel’s experience of treatment burden of DH in primary care, to understand the barriers and facilitators of the use of DH, and to map barriers identified to the Burden of Treatment Theory (BOTT).

**Design:**

A systematic literature review. MEDLINE, Psych INFO, EMBASE, and Cumulative Index to Nursing and Allied Health Literature (CINAHL) and Google Scholar will be searched. Two independent reviewers will screen papers using inclusion and exclusion criteria, with conflicts decided by a third reviewer. Any retrieved study that meets the inclusion and exclusion criteria will be quality appraised using the appropriate Critical Appraisal Skills Programme (CASP) checklist. The findings will be analysed using thematic synthesis and evaluated in the context of the Burden of Treatment Theory. The Preferred Reporting Items for Systematic Reviews and Meta-Analyses Protocol (PRISMA) guidelines have been adhered to in the production of this protocol.

**Conclusions:**

Understanding the experience of treatment burden whilst using DH in the military has the potential to influence health policy, the commissioning of services and interventions, and most importantly, improve patient experience and health outcomes. PROSPERO registration number: CRD42023494297.

## Introduction

### Rationale

As the global demand for healthcare continues to outstrip the supply of care, healthcare systems are beginning to explore the exploitation of digital health (DH) to meet the growing demand and optimise services by improving effectiveness, efficiency, and scalability ( King’s Fund, 2021; Kueper *et al.*, 2020).

DH refers to the intersection of digital technologies with healthcare to improve the overall efficiency and effectiveness of healthcare delivery, as well as improve patient experience ( Kingsfund, 2021). The integration of DH in primary care is widely accepted to have improved patient engagement, healthcare delivery, and overall patient outcomes. It is extensively used in military primary care as well as in wider general population health systems ( MOD, 2023; NHS, 2023a). It is possible that the use of DH modalities transfers work from health services to the patient, increasing their healthcare workload, which could potentially become burdensome.

Although DH is often viewed as convenient and efficient, it is crucial to gain a deeper understanding of the patient’s experience of treatment burden. Gaining this understanding will help inform how and what services are delivered and in what circumstances. Treatment burden encompasses the workload or demand placed on the patient as a consequence of managing their health ( Morris *et al*., 2021; Powell *et al*., 2021). These demands could be physical, emotional, financial, or psychological. Given the rise in global security risks, the military plays a crucial role in protecting human life and promoting peace and stability. Hence, it is necessary to consider the health needs of such an important demographic. An examination of the use of DH and the experience of treatment burden among military personnel is essential, as it plays a vital role in maintaining service personnel health and readiness through the promotion of preventative care, improved allocation, accessibility and availability of resources. Understanding treatment burden is critical for improving military health systems and overall patient outcomes by enhancing patient-centred care, treatment adherence, health equity, resource allocation, and people’s capacity to manage their health and treatment workload.

The military has adopted DH based on recommendations by the National Institute for Health and Care Excellence (NICE), which aligns with the National Health Service (NHS). Nevertheless, due to the unique nature of service life, there are marked differences between the civilian population and military personnel. In light of the ongoing evidence highlighting the increasing demand and scarcity of resources in healthcare, digitalisation has emerged as a means to efficiently scale and make evidence-based interventions cost effective and accessible ( EOHSP, 2021; Leightley & Murphy, 2022). An example is the use of DH to deliver psychological therapies, making them more widely available and accessible ( NHS, 2023).

Burden of Treatment Theory (BOTT) seeks to explore the impact of the complexity and demands of healthcare on the lives of patients and their support networks, and the resources required to manage health conditions and navigate health and social care systems. It considers the overall patient experience given the various tasks, time and efforts required to adhere to medical treatments, and manage health ( May *et al*., 2014). This review aims to explore the experience of treatment burden of DH for military personnel in primary health care. Applying BOTT will allow us to understand and address the difficulties and challenges faced by military patients using DH, with the aim of reducing the treatment burden and improving the patients’ quality of life ( May, 2005; May *et al*., 2014).

### Objectives

The purpose of this systematic review is to (1) understand the patient experience of the treatment burden of DH initiatives and interventions adopted by the military, (2) understand the barriers and facilitators to the use of DH, and (3) map the barriers identified to BOTT.

## Methods

This protocol has been produced in conformance with the Preferred Reporting Items for Systematic Review and Meta-Analysis Protocol (PRISMA-P) 2015 ( Shamseer *et al*., 2015) and is registered with PROSPERO (CRD42023494297).

Any identified treatment burdens will be connected to or associated with the following four components of BOTT: mobilising capacity, expressing capacity, mobilising for delegated tasks, and enacting delegated tasks. Any identified treatment burdens that are identified as being inconsistent with BOTT will be highlighted and commented on. Table 1 shows the summary of the inclusion and exclusion criteria.

**Table 1.** Summary of inclusion and exclusion criteria.

	Inclusion Criteria	Exclusion Criteria
Study Design	Qualitative and mixed-methods studies which have a qualitative element.	Quantitative studies.
Population	Regular serving armed forces personnel (Army, Navy, Marines, and Air Force), Gurkhas, Military Provost Guard Staff, mobilised Reservists and Full-Time Reserve Service personnel.	Studies that concern the treatment of veterans and civilian personnel employed by the military and non- military population groups.
Exposure	The full scope of digital health which includes, health information technology, telemedicine, telehealth, mobile health, digital medical devices, digital applications, software, and platforms that have been or are being used within primary care for diagnostics, treatment and/or self-care of any condition.	Assessments, diagnostics, and treatments not involving digital health. Digital health intervention used outside of the primary care setting.
Comparator	The review does not seek to make comparisons; however, if studies are retrieved that compare DH to conventional approaches, e.g., face-to-face, will be noted and commented upon.	N/A
Outcome	Patient experience of the treatment burden of DH in military personnel in primary care. Treatment burden identified will be classified and mapped to the BOTT framework as follows: mobilising capacity, expressing capacity, mobilising for delegated tasks, and enacting delegated tasks. Any identified treatment burdens that fall out with BOTT will be highlighted and commented on.	N/A
Publication Type	Published or unpublished full-text articles.	Opinions, theses, editorials, systematic reviews, and conference proceedings.
Language	English language.	Non-English language.
Date	Publication date must be between Jan 2000 and current date.	Publication date prior to Jan 2000.

### Sources of information

The following electronic databases will be searched: MEDLINE, PsychINFO, EMBASE, and Cumulative Index to Nursing and Allied Health Literature (CINAHL) and Google Scholar. The International Prospective Register of Systematic Reviews (PROSPERO) has been checked for ongoing and completed reviews exploring treatment burden in military personnel, and no such review was found. Ongoing monitoring of PROSPERO and Cochrane databases for similar reviews will be undertaken. Online search engines, government and military websites and library catalogues will be searched for grey literature. The search will be restricted to the timeframe from January 2000 to the current date, as the use of digital health prior to 2000 was limited due to technological limitations and centred on reactive care rather than preventative medicine. Since 2000, an abundance of technology combined with global health systems adopting preventative care, has led to the exponential growth of DH ( Abernethy *et al*., 2022).

### Search strategy

An information scientist at the University of Glasgow provided input to formulate the search strategy, which consists of the following concepts: digital health, military personnel, treatment burden, and patient experience. The search process queried the databases and exploited search features such as truncations, wildcards, and operators combined with Boolean terms. The search strategy used is summarised in Table 2 and can be found here: DOI: 10.5525/gla.researchdata.1546.

**Table 2.** Summary of search strategy terms.

Key Concept	Digital Health	Military Personnel	Treatment Burden
Search index terms and keywords	internet or web or online adj3 cognitive or behavio* or iCBT or i-CBT or ePsych* or e-Psych* or cCBT or c-CBT or home base* or digital technologies or geofenc* or beacons or nudge or place base* or wearable technology or wearable sensor or automated detection android or app or apps or blog* or CD-ROM or cell phone or cellphone or chat room or computer* or cyber* or digital or technology based or DVD or eHealth or e-health or electronic health or e-mail* or email* or e-Portal or ePortal or eTherap* or e-therap* or forum* or gaming or information technolog* or instant messag* or messaging or internet* or ipad or i-pad or iphone or i-phone or ipod or i-pod or podcast or smart phone or smartphone or social network* site* or social networking or mHealth or m-health or mobile or multi-media or multimedia or online* or on-line or personal digital assistant or PDA or SMS or social medi* or software or telecomm* or telehealth* or telemed* or telemonitor* or telepsych* or teletherap* or tele-health* or tele-med* or tele-monitor* or tele-psych* or tele-therap* or text messag* or texting or virtual* or web* or WWW or youtube or virtual or podcast blogging or e-mail or social media or text messaging or videoconferencing or webcast or wireless communication or telecommunication or teleconference or telemedicine or telehealth or telepsychiatry or teletherapy mobile phone or smartphone or mobile application or *technology or computer program or digital computer or personal computer or computer assisted therapy or *computer or telecomm* or tele- comm* or eLearning or blended learning or videoconferenc* or video conferenc* digital or technology based or eHealth or eTherap* or information technolog* or telehealth* or teletherap* or virtual* or internet or web or online or mental health screening	military or service person* or navy or air* or army or marine* or armed force*	experience, journey, or attitude* or attitude* to health treatment burden or burden of treatment or burden of care or quality of life or qol

### Study records

***Data management***. Database search results will be exported and downloaded to EndNote, where duplicates will be removed. The studies will then be uploaded to DistillerSR for screening by the reviewers. DistillerSR automates the management and screening of literature ( Dobbins *et al*., 2023).

***Selection process***. The screening process will be undertaken in three phases: title screening, abstract screening, and full text screening. The eligibility criteria will be agreed upon, and the title and abstract screening phases will be undertaken by the primary researcher (PE) and another member of the review team. Studies that meet the eligibility criteria will proceed to the full text screening phase. A full text screening will be conducted by PE and another member of the review team. Any disagreements will be resolved through discussion with a third reviewer from the study team.

***Data collection process***. The data extracted from the eligible studies will be used to populate a pre-agreed data extraction table and study characteristics table by PE and another member of the review team. Any disagreements will be resolved through discussion with the third reviewer.

### Data items for extraction

***Study characteristics***. Details such as the study aim, objective, design, setting, location, and period will be collected.

***Population***. Population characteristics, which include the following, will be collected: size, gender, age, ethnicity, nationality, rank, arm of service, duration of service, detail of duty station, and exposure to combat.

***Exposure***. The exposure in this study is the use of DH. A taxonomy of DH will be created to define DH in the context of this review. Variation in exposure in the different studies, duration of use, and user acceptability will be noted and commented on. Data collected will include details of the type and purpose of DH used.

***Comparator***. The review does not seek to make comparisons; however, if studies are retrieved that compare DH to conventional approaches, such as face-to-face, this will be noted and commented upon.

***Outcome***. The review will record patient experience of treatment burden of DH among military personnel within primary care.

### Outcomes and priorities

The primary outcome is the patient experience of burden of treatment mapped to the BOTT framework. Any treatment burden that is identified but falls outside of the BOTT framework will be noted and discussed.

### Risk of bias in individual studies

Risk of bias will be independently assessed by two reviewers and checked by a third. Any disagreements will be resolved through discussion with the third reviewer. The Critical Appraisal Skills Programme (CASP) tool for qualitative studies and the Mixed Methods Appraisal Tool (MMAT) will be used to assess quality ( CASP, 2014; Pluye *et al*., 2011).

### Data synthesis

Findings from the studies will be synthesised using thematic synthesis and mapped to BOTT. Thematic synthesis has been selected as it is a widely known and acceptable method of synthesising qualitative data where the intent is to understand content and context ( Thomas & Harden, 2008). It presents qualitative findings as analysable data, while also identifying themes ( Thomas & Harden, 2008).

Thematic synthesis will be broken down into three phases as follows: (a) line by line coding of the discussion and findings sections of the studies using NVivo software (version 14); (b) the development of overarching descriptive themes by consolidating the codes derived from phase ‘a’ into related areas; and (c) the development of analytical themes. Identified treatment burden themes will be linked to the four components of BOTT as follows: mobilising capacity, expressing capacity, mobilising for delegated tasks, and enacting delegated tasks. Data synthesis will be undertaken by PE and another member of the review team.

## Ethics approval and dissemination

Ethics approval is not required for the systematic review since it will not involve any identifiable patient data. However, the included studies will be checked to ensure that they have ethics approval as part of this review’s quality appraisal. The findings will be disseminated via conferences, peer-reviewed publications, and social media, and by presenting them to the Defence Medical Services Research Steering Group. This systematic review forms part of the primary researcher’s overall PhD project.

## Discussion

The aim of this systematic review is to evaluate all available literature exploring patient experience of treatment burden of DH initiatives and interventions adopted by the military in service personnel within primary care. Having undertaken scoping work with bibliographic databases, we believe this will be the first review to explore the treatment burden of DH among service personnel within primary care. The review expects to identify treatment burdens classifiable under the BOTT framework and to identify areas worthy of further research. This review will provide evidence to support the commissioning of new DH interventions and improve the capacity of health providers by highlighting the barriers, facilitators, and priorities of the use of DH. Additionally, it will help identify measures that will help improve patient capacity to cope with treatment burdens.

The review will be diligently undertaken which will be evidenced by strict adherence to PRISMA. The CASP and MMAT checklists are widely acknowledged tools for assessing the quality of the included studies ( CASP, 2014; Liberati *et al*., 2009; Pluye *et al*., 2011). The use of two reviewers at all stages of screening, data extraction, and quality appraisal will minimise bias, as will the checking of a proportion of coding by a second researcher and facilitating discussion of themes arising within the research team. Another strength is that the search strategy takes into account the definition of DH in a wide context, enabling the inclusion of a diverse range of studies. The data analysis will utilise pre-existing theory (BOTT) while also allowing flexibility for the inclusion of treatment burdens that fall outside of the remits of the framework.

## Challenges

Given that very few studies have explored the military patient’s experience of treatment burden, the availability of usable data remains a challenge. It is hoped that this challenge will be overcome by the rigorous and robust search strategy and the use of grey literature. As the data will be presented in various formats within the studies being reviewed, a challenge will be extracting, collating, and presenting the findings in a comprehensible and useful format. Another challenge is the potential for variations in interpretation among reviewers, even when a well-defined protocol is in place. Additionally, there is the potential for unforeseen issues or new insights influencing the methodology. Adopting a collaborative approach and discussing inconsistencies will help to overcome this.

## Conclusion

This review will shed light on the treatment burden challenges military personnel experience while using DH for primary care. The work will inform and guide future research and interventions in this evolving area of military healthcare research.

## Data Availability

University of Glasgow Enlighten Research Data. A systematic review of the experience of treatment burden of Digital Health for Military Personnel in Primary Health Care – Research Data. https://doi.org/10.5525/gla.researchdata.1546 This project contains the following underlying data: Data file 1. (Search strategy – Embase) Data file 2. (Search strategy – Medline, PsychInfo and CINAHL) PRISMA P checklist for ‘A systematic review of the experience of treatment burden of digital health for military personnel in primary healthcare – Research Data’. https://doi.org/10.5525/gla.researchdata.1546 Data are available under the terms of the Creative Commons Attribution 4.0 International license (CC-BY 4.0).

## Software availability

The software utilised in this research, Nvivo version 14, is available for purchase through the official website at: Nvivo 14. Additionally, a free trial can be accessed for a period of 14 days. Researchers interested in trying the software can visit Nvivo 14 free trial to initiate the trial.

For further information, support and updates regarding Nvivo version 14, please refer to the official website of QSR International. Please note that the links provided may be subject to change, and users are encouraged to visit the official website for the most up to date information.
