# Supplementary material for: A systematic review of the experience of treatment burden of digital health for military personnel in primary healthcare
Source: Health Open Res. 2024 Mar 8;6:9. [Version 1] doi: 10.12688/healthopenres.13599.1 (PMC11638906; doi:10.12688/healthopenres.13599.1)
Supplement: Peer review history

# Reviewer report: Reviewer response for version 1

**DOI:** 10.21956/healthopenres.14686.r29059

**Published:** 2024-11-26

**Authors:** Jay A. Gorman

**Affiliations:** 1 VA Bedford Healthcare System, Bedford, Massachusetts, USA

**Comment:**

 This describes a protocol to conduct a systemic review to understand military personnel’s experience of treatment burden of Digital Health (DH) in primary care, specifically to understand the barriers and facilitators of the use of DH, and to map barriers identified to the Burden of Treatment Theory (BOTT). Overall, it is succinct and clear. Some comments below:

**1. Introduction: **Authors could describe BOTT in a bit more detail to better inform the reader and then elaborate on how BOTT will inform coding and if examples may benefit the reader.

**2. Method:** This would benefit from a clarification around the coding process, perhaps explaining in more detail about how the third reviewer will play a role in coding outside of “disagreements”, and how many items or percentage of items may be coded by both coders and checked by a third reviewer.

Is the study design appropriate for the research question?

Yes

Is the rationale for, and objectives of, the study clearly described?

Yes

Are sufficient details of the methods provided to allow replication by others?

Yes

Are the datasets clearly presented in a useable and accessible format?

Yes

Reviewer Expertise:

Veteran-centered care.

I confirm that I have read this submission and believe that I have an appropriate level of expertise to confirm that it is of an acceptable scientific standard.

# Reviewer report: Reviewer response for version 1

**DOI:** 10.21956/healthopenres.14686.r28180

**Published:** 2024-06-19

**Authors:** Shumenghui Zhai

**Affiliations:** 1 University of Washington, Seattle, USA

This protocol presents a promising approach for conducting a systematic literature review on the burden of DH. The unique population of the military brings a critical need for further understanding. Here are some constructive suggestions for improvement:

1. Consider clarifying in the abstract that the review focuses on the burden experienced by patients. It's important to specify the target population for the review and evaluation.
2. It would be beneficial to address any time frame or geographical limitations for the review. If there are no such limitations, providing a rationale for this decision would enhance the protocol.
3. Please provide more details on how the review results will be aligned with the BOTT framework. Clarity on this alignment will strengthen the overall approach.

Is the study design appropriate for the research question?

Yes

Is the rationale for, and objectives of, the study clearly described?

Yes

Are sufficient details of the methods provided to allow replication by others?

Yes

Are the datasets clearly presented in a useable and accessible format?

Yes

Reviewer Expertise:

digital health, sleep, caregiver, systematic review, qualitative research.

I confirm that I have read this submission and believe that I have an appropriate level of expertise to confirm that it is of an acceptable scientific standard.
